# The complete mitogenome of *Lasioglossum affine* (Hymenoptera: Halictidae) and phylogenetic analysis

**DOI:** 10.1080/23802359.2020.1835573

**Published:** 2020-11-20

**Authors:** Jinyi Wei, Huanhuan Lu, Jingxin Tao, Youjin Hao, Dunyuan Huang, Bo Li

**Affiliations:** a Chongqing Key Laboratory of Vector Insects, Chongqing Normal University, Chongqing, China; b College of Life Sciences, Chongqing Normal University, Chongqing, China

**Keywords:** *Lasioglossum affine*, Hymenoptera, mitogenome, phylogeny

## Abstract

The complete mitogenome of *Lasioglossum affine* (Hymenoptera: Halictidae) was sequenced and analyzed. The whole mitogenome is 17,352 bp (AT%=84.1%) and encodes 37 typical eukaryotic mitochondrial genes, including 13 protein-coding genes (PCGs), 22 *tRNAs*, two *rRNAs*, and an AT-rich region. Further analysis found three gene rearrangements, where *trn I-Q-M* → *trn M-I-Q*, *trn W-C-Y* → *trn C-W-Y*, and *trn K-D* → *trn D-K* were shuffled. The phylogenetic relationships of 19 species of Hymenoptera were established using maximum-likelihood method based on 13 concatenated PCGs. The result showed that *Lasioglossum affine* is a sister of *Lasioglossum* sp. SJW-2017.

*Lasioglossum affine*, also known as *Evylaeus affine* (Smith 1853), belongs to genus *Lasioglossum* of the family Halictidae (Sakagami et al. 1982). It is a native species in Heilongjiang, Taiwan, Hunan of China, and also distributes in Japan, South Korea, North Korea, and Russia (Sakagami et al. 1982; Packer 1991; Murao et al. 2014). Up to now, little systematic research on the genus *Lasioglossum* in China has been conducted. Therefore, this gap needs to be addressed (Zhang et al. 2012). To explore the genus phylogeny, the first complete mitogenome of *L. affine* was sequenced and analyzed. The specimen was collected for the first time in Longping village, Cili county, Hunan of China (N 29.450217, E 110.096235). The samples used in this study are stored in the Key Laboratory of Vector Insects in Chongqing Normal University (No. 2018-HN-807). GenBank accession number of this mitogenome is MT780494.

A pair-end (PE) library was built and mitochondrial DNA sequencing was implemented on Illumina Nova seq 6000 platform (Illumina, San Diego, CA). Subsequently, *de novo* assembly and annotation of complete mitochondrial sequence were carried out using SPAdes v3.9.0 (Bankevich et al. 2012) and MITOS (http://mitos.bioinf.uni-leipzig.de/index.py) (Bernt et al. 2013), respectively. Finally, the complete mitochondrial genome of *L. affine* was assembled as a circular DNA molecule of 17,352 bp, encoding 13 protein-coding genes (PCGs), 22 *tRNAs*, two *rRNAs*, and an AT-rich region (also called control region, CR). For whole mitogenome, AT content is 84.1%. Compared with the ancestral sequence of Chalcidoidea (Hymenoptera) (Zhu et al. 2018), three gene rearrangements were found: *trn I-Q-M* → *trn M-I-Q*, *trn W-C-Y* → *trn C-W-Y*, and *trn K-D* → *trn D-K*. Additionally, nine overlapping regions between gene sequences were scattered throughout the whole mitochondrial genome.

Four types of start codons of 13 PCGs are used: ATA (*cox2*, *nad5*, *cytb*, and *nad1*), ATT (*cox1*, *cox3*, *nad3*, *nad4l*, and *nad6*), ATC (*nad2* and *atp8*), and ATG (*atp6* and *nad4*). The stop codon of all PCGs is TAA. Except for *trnS2*, all tRNA genes have a typical clover structure and the dihydrouridine (DHU) arm forms a simple loop, which is also conserved in other Hymenoptera species (Wei et al. 2010; Huang et al. 2016; Lu and Huang 2020). The 16S rRNA (*rrnL*) (1394 bp) and 12S rRNA (*rrnS*) (842 bp) genes are located on the light strand. The AT-rich region is 2311 bp.

To explore the phylogeny of *L. affine* in order Hymenoptera, 18 complete mitogenome sequences were downloaded from GenBank, including three outgroups from family Vespidae (*Vespa velutina*, *Vespa mandarinia*, and *Abispa ephippium*). The phylogenetic tree was constructed using 13 concatenated PCGs based on the optimized evolutionary model of GTR + G+I, and the maximum-likelihood (ML) tree was built using MEGA7 with 1000 bootstrap replicates (Kumar et al. 2016).

As shown in Figure 1, the phylogenetic relationships of all tested species were consistent with previous studies (He et al. 2018; Lu and Huang 2020). *L. affine* was a sister of *Lasioglossum sp. SJW-2017*. Taken together, the newly sequenced mitochondrial genome of *L. affine* can provide DNA barcodes for phylogenetic and evolutionary analysis among other insect species.

**Figure 1.**
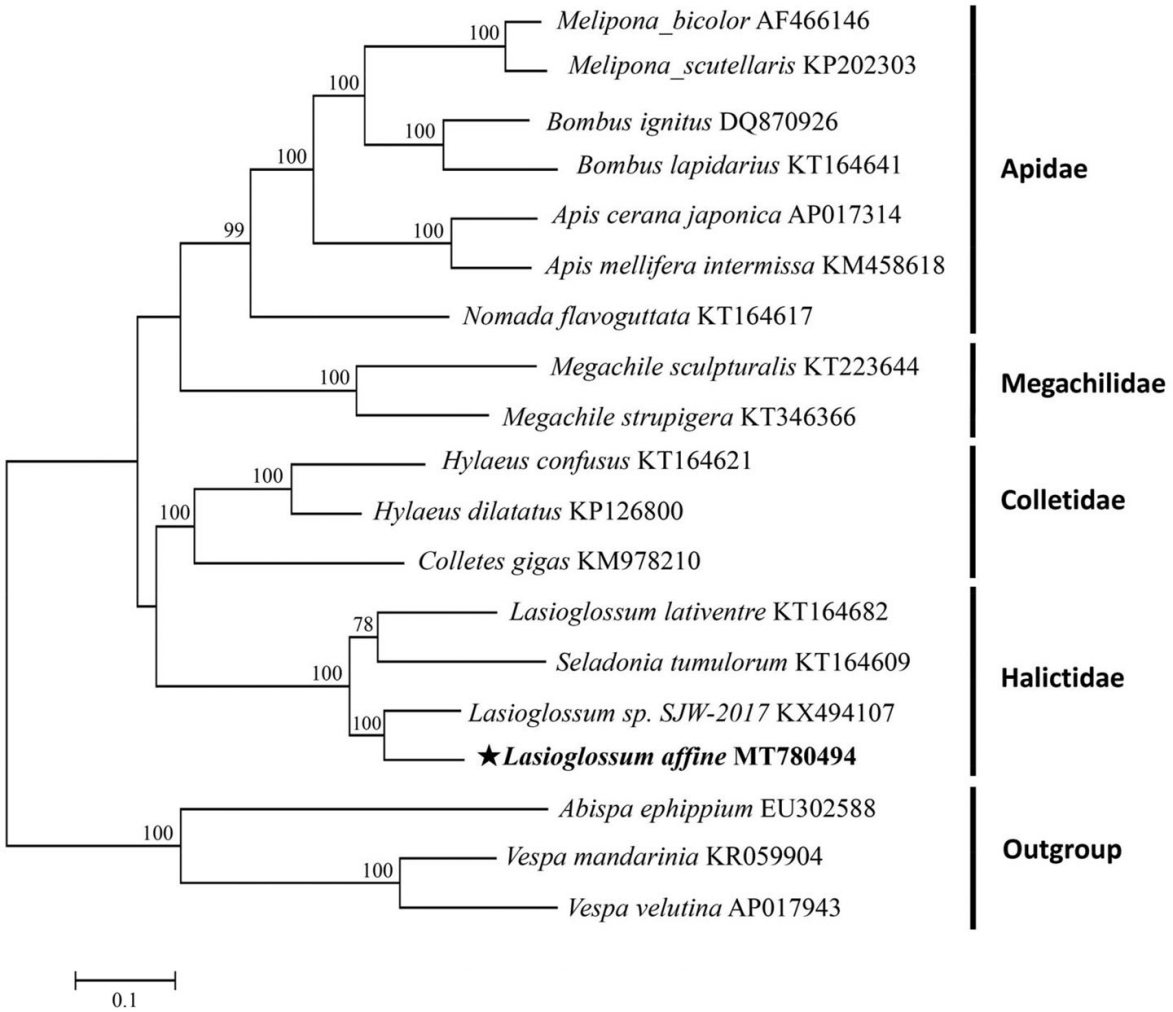
Phylogenetic tree of *L. affine* was constructed based on 13 PCGs of Hymenoptera species and three outgroups. The support values on the nodes indicate the percentages of 1000 bootstrap replicates. GenBank accession numbers were followed after their appropriate species names.

## Data Availability

The mitochondrial genome sequence used in this study can be obtained on NCBI through GenBank No. (NCBI https://www.ncbi.nlm.nih.gov/), and raw data were also openly available at SRA (https://trace.ncbi.nlm.nih.gov/Traces/sra/?study=SRP278000).
